# Set-theory based benchmarking of three different variant callers for targeted sequencing

**DOI:** 10.1186/s12859-020-03926-3

**Published:** 2021-01-07

**Authors:** Jose Arturo Molina-Mora, Mariela Solano-Vargas

**Affiliations:** 1 Centro de Investigación en Enfermedades Tropicales (CIET) and Facultad de Microbiología, Universidad de Costa Rica (UCR), San José, Costa Rica; 2 Centro de Investigaciones en Hematología y Transtornos Afines (CIHATA), Universidad de Costa Rica (UCR), San José, Costa Rica

**Keywords:** Variant calling, Isaac, Freebayes, VarScan, Set-theory, Inherited cardiac conditions, Next-generation sequencing

## Abstract

**Background:**

Next generation sequencing (NGS) technologies have improved the study of hereditary diseases. Since the evaluation of bioinformatics pipelines is not straightforward, NGS demands effective strategies to analyze data that is of paramount relevance for decision making under a clinical scenario. According to the benchmarking framework of the Global Alliance for Genomics and Health (GA4GH), we implemented a new simple and user-friendly set-theory based method to assess variant callers using a gold standard variant set and high confidence regions. As model, we used TruSight Cardio kit sequencing data of the reference genome NA12878. This targeted sequencing kit is used to identify variants in key genes related to Inherited Cardiac Conditions (ICCs), a group of cardiovascular diseases with high rates of morbidity and mortality.

**Results:**

We implemented and compared three variant calling pipelines (Isaac, Freebayes, and VarScan). Performance metrics using our set-theory approach showed high-resolution pipelines and revealed: (1) a perfect recall of 1.000 for all three pipelines, (2) very high precision values, i.e. 0.987 for Freebayes, 0.928 for VarScan, and 1.000 for Isaac, when compared with the reference material, and (3) a ROC curve analysis with AUC > 0.94 for all cases. Moreover, significant differences were obtained between the three pipelines. In general, results indicate that the three pipelines were able to recognize the expected variants in the gold standard data set.

**Conclusions:**

Our set-theory approach to calculate metrics was able to identify the expected ICCs related variants by the three selected pipelines, but results were completely dependent on the algorithms. We emphasize the importance to assess pipelines using gold standard materials to achieve the most reliable results for clinical application.

## Background

Next-Generation Sequencing (NGS) technologies and applications, including whole genome, whole exome, and targeted sequencing, have drastically improved the study of hereditary diseases. The field of NGS technologies is evolving rapidly with constant improvements. Hence, it is mandatory to benchmark bioinformatic pipelines to evaluate reproducibility with standardized protocols [24]. Several studies have shown that variant identification is influenced by complexity of genomic region, sequencing technology platform (including libraries and other preparation steps), and bioinformatics pipelines for data analysis [5, 9, 22, 23, 28]. In addition, performance testing is currently challenging due to the paucity of standards and the no consensus definition for performance metrics [9, 14]. Thus, pipelines can result in dramatic differences that affect medical decisions in clinical laboratories that are developing or relied on sequencing-based tests [9]. This may involve changes in the diagnosis, prognosis, and treatment of patients [20].

Accordingly, robust and standardized benchmarking methods are critical for the development, optimization, and comparison of sequencing, mapping, and variant calling tools [14]. In this field, benchmarking of variant calling methods is not straightforward [9]. Therefore, the gold standard and quality control materials are essential for the performance evaluation of approaches in both, experimental assays and data analysis pipelines. Efforts to create gold standard materials in order to compare NGS approaches have been developed by several programs, e.g. the Genome in a Bottle (GIAB) [31], Illumina Platinum Genomes Project, and Genetic Testing Reference Materials (GeT-RM) Coordination Program by NCBI [10]. Global Alliance for Genomics and Health (GA4GH) Benchmarking Team has also implemented the best practices guideline to standardize variant calling benchmarking [14].

In the particular case of the GIAB consortium, a reference material (high confidence genotype) for the pilot genome NA12878 (one individual sample in the 1000 Genome project) was developed to offer a benchmark set of small variants and reference variant calls [32]. This gold standard material has been used in different benchmarking studies of variant calling pipelines [5, 9, 25].

In this context, and as an example of a clinical application, we conducted a systematic comparison of three variant calling methods using NA12878 targeted sequencing data and the gold standard variant calls set. The recently suggested benchmarking framework of GA4GH was used as a reference to implement a new simple and user-friendly strategy to compare pipelines using a set-theory approach. Publicly available sequencing data was generated employing the TruSight Cardio kit (Illumina), a panel of 174 critical genes related to Inherited Cardiac Conditions (ICCs), i.e., a group of cardiovascular diseases with a genetic basis. ICCs such as cardiomyopathies, arrhythmias, aortopathies, and others [12] may have severe clinical manifestations, with high rates of morbidity and mortality [34]. A more detailed description and genes associated with some of these cardiopathies are presented in Additional file 1 “Analysis of variants related to ICCs”.

Thereby, identification of causative or related mutations in multiple ICCs-genes makes molecular biology and subsequent bioinformatics analysis key tools for confirming the diagnosis [34], studying overlapping phenotypes, patient management and prognosis, screening for family members, and other applications [20].

Altogether, our study aimed to assess a new simple method based on set-theory for benchmarking three variant calling pipelines for targeted sequencing data using gold standard material as a reference. To address this, NA12878 sequencing data from the TruSight Cardio kit was used to compare Isaac [21], Freebayes [7], and VarScan [13] pipelines. To contrast identified variants by callers against gold standard data, we implemented a new simple method based on set-theory. This method represents an easy and user-friendly approach to evaluate custom implementations in variant calling analysis.

## Results

### A general comparison between pipelines

**Fig. 1:**
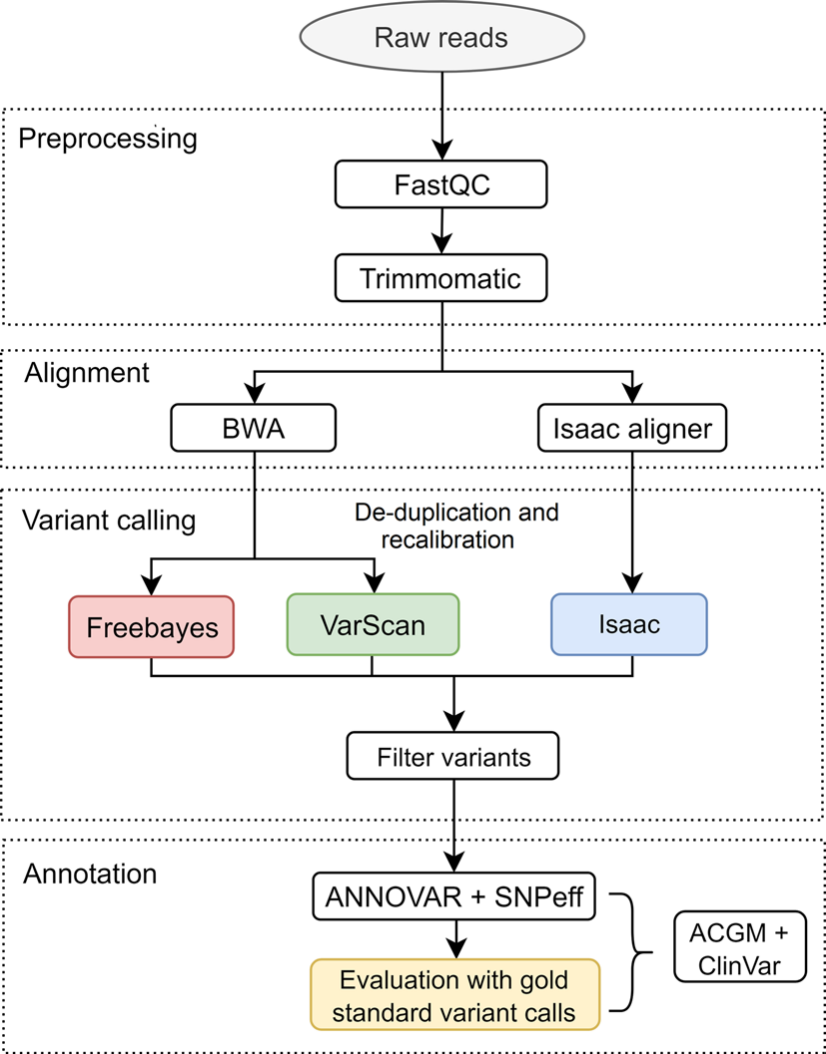
Workflow for variant calling analysis by three pipelines

**Fig. 2:**
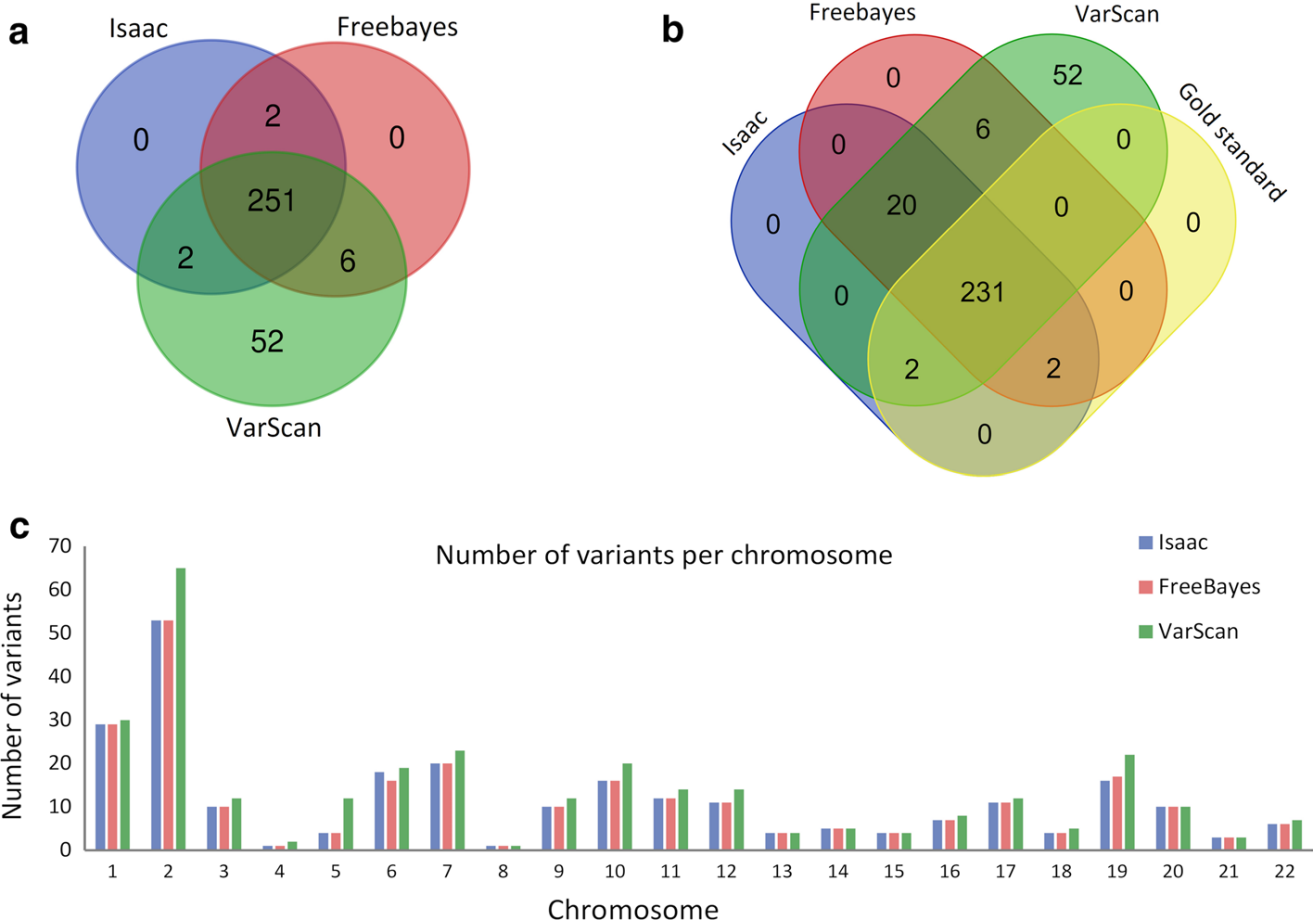
Comparison of identified variants by three variant calling pipelines (**a**), including benchmark using the gold standard variants set (**b**) and the number of variants per chromosome (**c**)

In order to evaluate the performance of three variant callers using targeted sequencing data, Isaac, Freebayes, and VarScan pipelines were implemented (see Figs. 1 and 2). A different number of total variants (SNP) were obtained with the three pipelines: 255, 259, and 311 for Isaac, Freebayes, and VarScan, respectively. The ratio of transitions/transversions (Ts/Tv ratio), variant rate, and functional class were similar in all the cases, being the VarScan approach the one that differed the most (Table 1).

**Table 1:** General metrics for three variant calling pipelines

Metrics	Variant calling pipeline		
	Isaac	Freebayes	VarScan
Number of variants (SNP)	255	259	311
Variant rate	1/1 121 0146	1/11 633 348	1/9 799 651
Ts/Tv			
Transitions (Ts)	204	208	241
Transversions (Tv)	51	51	70
Ts/Tv ratio	4.00000	4.07841	3.4429
Functional class			
Missense	218	237	321
Silent	495	499	507
Missense/silent ratio	0.4404	0.4749	0.6331

Besides, 251 common variants were identified by the three algorithms (Fig. 2a), while 52 exclusive variants were found with VarScan. When we compared the variants obtained by the three pipelines with the gold standard set, 231 true variants were identified (Fig. 2b). Performance comparison in the number of variants identified per chromosome revealed that Freebayes and Isaac had a similar resolution, which contrasted with slightly higher values obtained by VarScan (Fig. 2c).

### A set-theory approach for evaluating variant calling pipeline performance

**Fig. 3:**
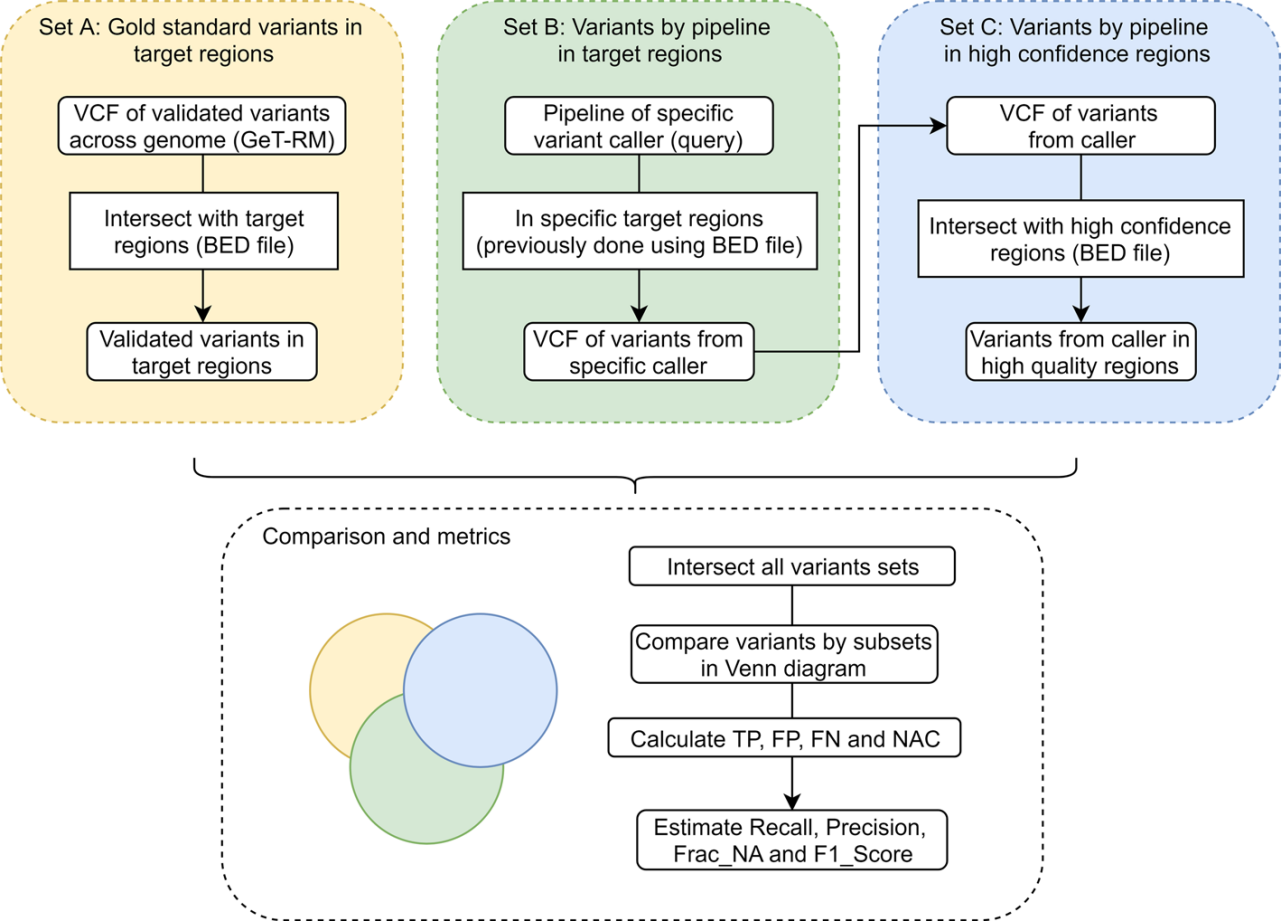
Set-theory approach for benchmarking variant calling pipelines

Using the gold standard variants and high confidence regions of the NA12878 sample as a template, we implemented a simple and user-friendly approach using the set-theory methodology. The final model was achieved with the following sets (more details are presented in Fig. 3):

- *Set A*: List of all variants in gold standard variant calls in targeted sequencing regions.
- *Set B*: List of all identified variants by pipeline in targeted sequencing regions.
- *Set C*: List of all identified variants by pipeline in high confidence genomic regions.

Each set was created using the variant calling files (VCF, for both gold standard material and evaluated pipeline) and the chromosomal regions (BED file for both targeted sequencing genes and high confidence material). A total length of 271 227 bp was obtained intersecting target regions (total length 575 148 bp) and high confidence regions. This means that 47.2% of the target regions are part of high confidence regions.

**Fig. 4:**
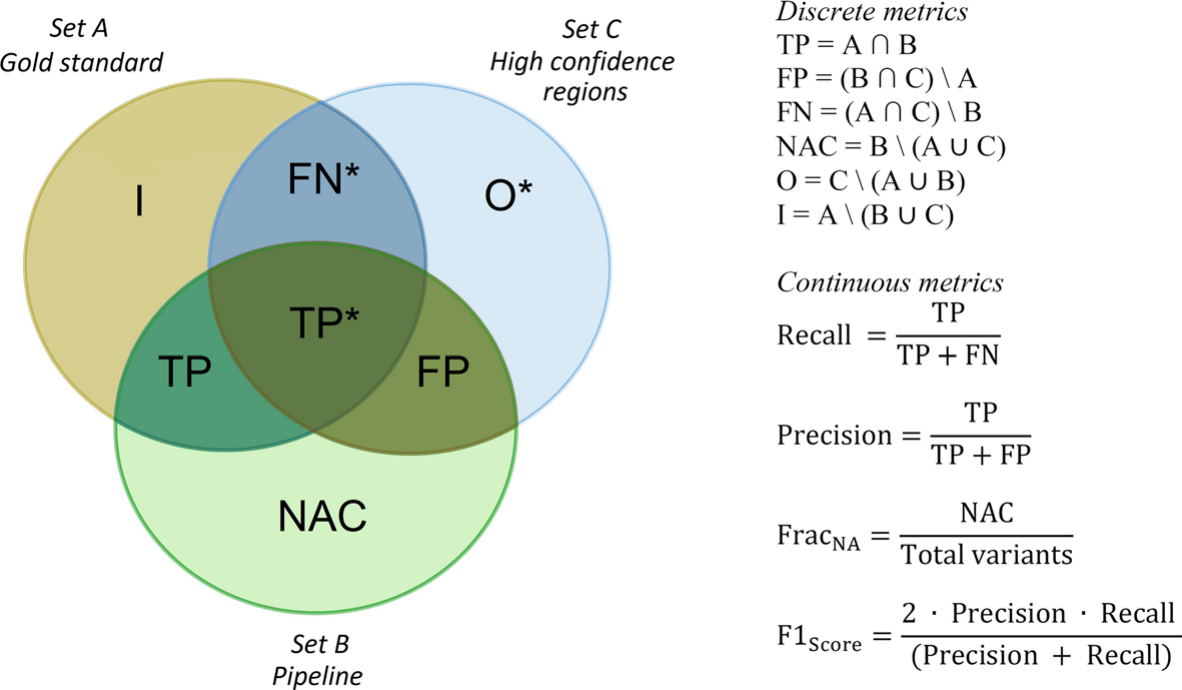
Metrics calculation for evaluation and validation of variant calling pipelines using a set-theory model (true variants in the high confidence regions are marked with *)

Our set-theory approach can be used not only to identify a list of variants in each case but also to calculate metrics. Metrics performance was defined, as explained below, either by using basic set operations according to set-theory mathematics (discrete metrics, value is an integer number) or by using ratios (continuous metrics, value is a real number) (see details in Fig. 4).

### Discrete metrics: based on set-theory operations

- True positives (TP): variants called by a variant caller as the same genotype as the gold standard data, also known as analytical sensitivity. TP is calculated by intersecting sets A and B: TP = A ∩ B
- False positives (FP): variants called by a variant caller but not within the gold standard variant set. In this approach, it is calculated by intersecting sets B and C, and then eliminating variants in gold standard material: FP = (B ∩ C)\A. Variants in high confidence region are reported in benchmark variants set, thus, an identified variant absent in gold standard set is one FP.
- False negatives (FN): gold standard variants in high confidence regions that were not called by the variant caller pipeline: FN = (A ∩ C)\B
- Non-assessed calls (NAC): variants outside the high confidence regions or gold standard material: NAC = B\(A ∪ C)
- Out of region of interest (O): variants in high-quality regions out of target regions. Not really useful for pipelines evaluation by targeted sequencing, but important for developers of gold standard materials in other contexts: O = C\(A ∪ B)
- Incongruences or Discordances (I): gold standard variants outside the high confidence regions that resulted not called by the variant caller pipeline: I = A\(B ∪ C)
- True Negatives (TN): Due to limitations related to current reference materials, which makes TN difficult to interpret, we did not include it [14].

### Continuous metrics: ratios

- Recall or sensitivity: ability to detect variants that are known to be present, i.e., the absence of FN. It is calculated as: $\mathrm{Recall }=\frac{\mathrm{TP}}{\mathrm{TP}+\mathrm{FN}}$
- Precision or specificity: ability to correctly identify the absence of variants, true negatives, or the absence of FP: $\mathrm{Precision }=\frac{\mathrm{TP}}{\mathrm{TP}+\mathrm{FP}}$
- ${\mathrm{Frac}}_{\mathrm{NA}}$: Proportion of non-assessed calls by a pipeline or not included in the reference materials. It is calculated using NAC: ${\mathrm{Frac}}_{\mathrm{NA}}=\frac{\mathrm{NAC}}{\mathrm{Total\,variants}}$
- ${\mathrm{F}1}_{\mathrm{Score}}$ or F-measure: This metric integrates precision and recall in a single value using the harmonic average: ${\mathrm{F}1}_{\mathrm{Score}}=\frac{2 \cdot \mathrm{ Precision }\cdot \mathrm{ Recall }}{(\mathrm{Precision }+\mathrm{ Recall})}$

**Fig. 5:**
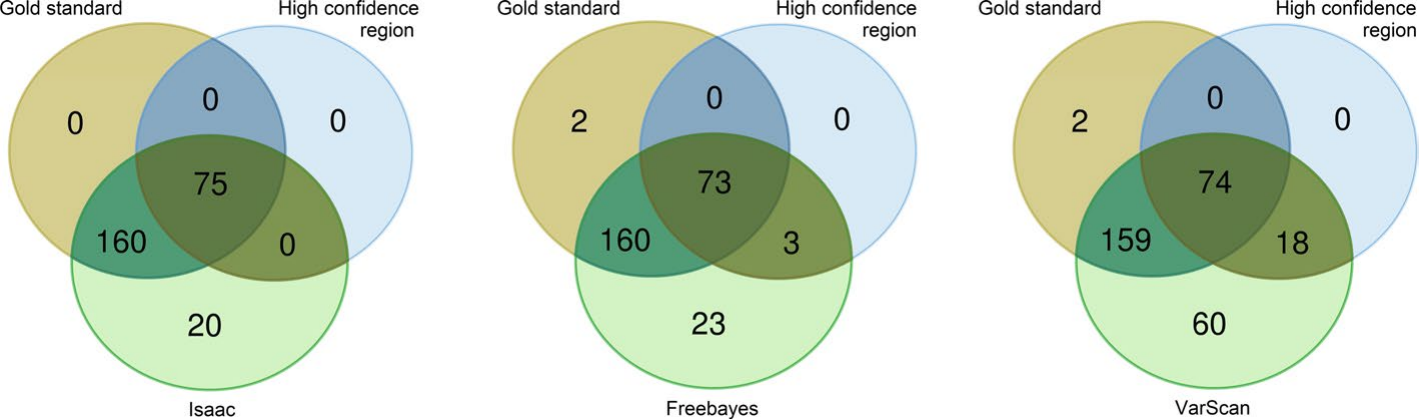
Set theory approach to benchmark three variant calling pipelines

We applied our set-theory approach to the TruSight Cardio data set. We analyzed data in each pipeline to contrast the results against high confident regions and the gold standard set. Comparison between gold standard in the high confidence region and each pipeline found 73 true calls for Freebayes, 74 for VarScan, and 75 for Isaac. VarScan had more NAC variants (60) compared to the other two pipelines (Isaac: 20, Freebayes: 23) (Fig. 5). All metrics were calculated according to definitions, and next, hap.py pipeline was applied to the Isaac results using the same gold standard set. As expected, all metrics found by both approaches, i.e., our set-theory model and the hap.py pipeline, rendered exactly the same results. Table 2 shows that 233 TP were identified for VarScan and Freebayes, and 235 for Isaac. None FP was recognized for Isaac, only three for Freebayes, but 18 for VarScan.

**Table 2:** Evaluation of metrics for pipelines validation in high quality regions

Variant caller	Metrics								
	TP	FP	FN	I	NAC	Recall	Precision	Frac_NA	F1_Score_
Freebayes	233	3	0	2	23	1.000	0.987	0.089	0.989
VarScan	233	18	0	2	60	1.000	0.928	0.193	0.959
Isaac^a^	235	0	0	0	20	1.000	1.000	0.078	1.000

^a^Same values were obtained when hap.py pipeline was run

Recall was 1.000 for all pipelines, and precision rates were 0.928 for VarScan, 0.987 for Freebayes, and 1.000 for Isaac. No FNs were identified, but two incongruent variants (I) were found for Freebayes and VarScan. Thus, VarScan showed slightly lower performance, as reflected by the global performance value ${\mathrm{F}1}_{\mathrm{Score}}$.

**Fig. 6:**
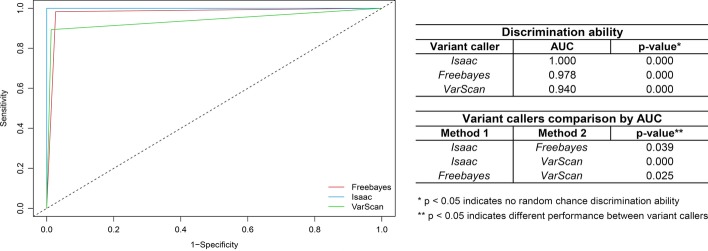
Receiver operating characteristic (ROC) curve to assess and compare the variant callers

For the three pipelines, ROC curves presented good performance, with AUC > 0.94 in all cases (p = 0.000), including perfect AUC = 1 for Isaac approach (Fig. 6). When pipeline comparison was done using AUC values, a statistically significant difference (p < 0.05, Fig. 6) was encountered in all the cases. In general, the results indicate that the three pipelines can discriminate the variants, with differences between algorithms.

Finally, we selected all identified variants by the three pipelines in ten HMC related genes. These variants were annotated using ClinVar and InterVar, and none was found to be pathogenic. They were classified mainly as benign or likely benign using the NA12878 sample at genomic regions of the targeted sequencing. See Additional file 1: Table S2 “Analysis of variants related to ICCs” for details of the SNPs, genes, and SNP classification by ACGM and ClinVar. This material also includes an analysis of false positive and incongruent variants (Additional file 1: Table S3).

## Discussion

One of the challenges of analysis in bioinformatics is dealing with the complexity of the data and the variable results, which depends in a great matter on algorithms and parameters [22]. Since high-throughput targeted sequencing is now used as part of the clinical routine [22] chmarking of bioinformatics pipelines is required to better understand the accuracy of sequence data, identify underlying causes of error, and quantify the improvements obtained from algorithmic developments for its use in clinical settings [6, 29].

In our study, three variant calling pipelines were evaluated. Freebayes employs a Bayesian statistical framework using a haplotype-based approach [7], meanwhile, VarScan uses a robust heuristic/statistic model [13]. In the case of Isaac Enrichment, it is a consensus approach that combines both Bayesian and heuristic analysis [21]. Diversity in the variant caller algorithms can explain the variable results in pipeline performance. For example, 311 variants were identified with VarScan, which contrasts with the 255 and 259 variants identified with Isaac and Freebayes, respectively. Accordingly, a higher dissimilarity was observed in type and impact general metrics for VarScan, than for Isaac and Freebayes. This profile between variant callers was observed not only for the discrete and continuous metrics but also for Ts/Tv ratio, a value commonly used as quality control. Some studies indicate that SNPs in exome regions should have a Ts/Tv ratio around 3, emphasizing that increment in Ts/Tv ratio usually indicates better quality [26].

As in our case, due to substantial discordance among callers, NGS strategies require highly accurate reference materials to benchmark bioinformatic pipelines [31, 32]. Nevertheless, comparing variant calls from a particular sequencing pipeline to a gold standard set is not a trivial task, since there are different representations of the variants, the definitions for performance metrics are not yet standardized and the performance can vary across variant types and genomic region [14]. As discussed in recent studies and suggested by best practices for benchmarking variant callers, interpretation of different metrics (including FN and TN) is conflictive due it depends on the reference materials, the definition (i.e. the way to calculate it), and the algorithms that are applied [14, 33]. Thus, robust and standardized strategies are required to compare the performance of variant callers.

In this sense, we implemented a user-friendly and simple method using a set-theory approach to benchmark variants against a gold standard. Although there are automatic packages for the calculation of metrics and subsets of lists, our workflow based on a model of set-theory allows simple management for users in terms of:

1. Understanding the logic and comparison strategy between variants of gold standard sets, high confidence regions, and the variants identified by pipelines. This differentiates it from automatic methods because this aspect usually is not explicitly shown to users. In this way, congruencies/inconsistencies between sets can be determined visually.
2. Variants lists are obtained according to each set or subset, thus variants can be described and annotated specifically under any of the conditions (e.g. variants falsely detected as true or negative). Other subsets of interest (variants outside the region of high confidence, variants not evaluated, incongruences, etc.) are easily identified.

In addition, since we focused on the benchmarking of variant callers and not on the particular results (using the NA12878 sample at genomic regions of the targeted sequencing as an example), our approach is flexible since it can be used to test newer versions of the gold standard set, other high confidence intervals, different target regions, and other variant callers.

With our approach, general metrics suggested a similar performance between Freebayes and Isaac, meanwhile, VarScan detected more variants classified as FP. These results are consistent with the ROC analysis for all pipelines, although VarScan showed a slight reduction in discriminatory ability. Besides, the comparison by AUC showed a statistically significant difference in the performance between all three pipelines. Some authors have found that Freebayes has the highest recall but lower accuracy compared to the other algorithms [6], while others associate better capacity with Isaac [20]. Our results are similar to the findings of Cheng et al. [3], which obtained less precise results using VarScan when comparing other variant callers [3]. Conversely, in some studies, VarScan has performed well compared to other algorithms [30].

In the context of ICCs and genomic analysis, our results can provide a practical and comprehensive guide to evaluate variant calling pipelines and select the best options for analysis of targeted sequencing data, as shown in the case of the TruSight Cardio kit. Under this scenario, a clear contribution can be made to give accurate and high-quality information during clinical decisions. Other targeted sequencing data can be used with our approach in a similar way.

On the other hand, we emphasize that reference materials are important not only for benchmarking variant calls, but also to stratify performance by variant type and genome context, including the case of novel variants, that not always are present in gold standard sets [14]. Another limitation is the use of a single data set, as in our case, therefore results must be taken with caution [11].

Altogether, it is anticipated that genomic sequence information will continue improving the clinical diagnosis as part of the new initiatives in precision medicine [6]. However, further analyses to optimize benchmarking bioinformatics pipelines are still required.

## Conclusions

We implemented and compared the variant calling pipelines Isaac, Freebayes, and VarScan using targeted sequencing data. Benchmarking was done using reference materials and a new simple and user-friendly strategy based on set-theory for metrics calculation and results analysis. As expected, results were completely dependent on the selected algorithms, but in all cases with a good discriminatory ability to identify variants. Isaac and Freebayes pipelines had comparable performance, while VarScan resulted in more dissimilar results. These differences evidenced that there is a gap in respect to the implementations of standard pipelines and that there is still a dependence on the nature of the data and the general experimental design. Therefore, it is still necessary to benchmark pipelines for specific data and characterize the conditions in which the most reliable results are achieved, which is critical in clinical applications.

## Methods

### Data source

Targeted sequencing data (fastq files, using TruSight Cardio kit) of reference genome NA12878 was retrieved from the BaseSpace public database. Data were generated using paired-end sequencing in a MiniSeq instrument using 2 × 76 bp read length [20].

Data and details: https://basespace.illumina.com/sample/35737707/NA12878. Access by the web-tool or command-line requires an account (free) in the BaseSpace platform. To access by command-line, see instructions here: https://bioinformatics.uconn.edu/data-download-from-basespace-illumina/.

### Pre-processing: quality analysis

Data quality evaluation was done with FastQC (version 0.11.6) using a standard analysis [1] including base quality, GC content, and sequence length distribution. Selection of reads for removing adapters and low-quality bases (Q < 20) was done using Trimmomatic (version 0.36.3) [2], and reviewed by FastQC again.

### Read alignment and variant calling

In order to identify single nucleotide polymorphisms (SNPs), three variant calling pipelines were implemented and evaluated (pipelines were named according to variant calling algorithms: Freebayes, VarScan, and Isaac). An overview of the general protocol employed is shown in Fig. 1. The same files were processed with the three pipelines.

To align reads to the reference human genome (GRCh37-hg19), BWA (version 0.7.17.4) alignment package was run using default parameters [16]. for Freebayes and VarScan pipelines. Picard tools (version 2.18.0) were used to remove eventual duplicate reads from single fragments of DNA [28]. For Isaac, the pipeline was run in the BaseSpace platform (https://basespace.illumina.com, which includes its aligner; fastq files were incorporated directly to map and then run the variant calling.

For the variant calling step (after alignment, Freebayes (version 1.0.2–29) [7], VarScan (version 0.1) [13] and Isaac [20, 21] algorithms were implemented with default parameters.

Variants filtering was done using VCFfilter [18] (Version 1.0.0, default values for other parameters), including depth/coverage (DP > 10) and quality (QUAL > 20).

### Annotation of variants and pipelines comparison

The VCF files of the three pipelines were annotated with ANNOVAR (version 0.2) [27]. using refGene as reference for gene annotation and snp137 as annotation database [27, 28]. Functional effect prediction was evaluated running SNPeff (Version 4.3) [4].

### Pipelines performance against a gold standard: a set-theory approach

Benchmark of variant callers was done against high confidence regions and gold standard variants set (NIST v2.18) for SNPs in NA12878. Both data set were downloaded from the GeT-RM Browser (https://www.ncbi.nlm.nih.gov/variation/tools/get-rm/).

To compare variant calling pipelines, a user-friendly and simple method based on a set-theory approach was implemented. An exhaustive evaluation for building variant lists (sets) was done to establish variant callers performance. The evaluation included the following materials: (1) list of variants of a particular pipeline using a specific caller, (2) targeted sequencing regions, (3) high confidence regions of NA12878 vs reference genome (for analytical specificity evaluation), and (4) gold standard variants of NA12878 (for analytical sensitivity evaluation). Variants were obtained in VCF files and BED files-regions.

The materials were used to create the final set conformation of the model, which was defined using set operations and ratios along with mathematical expressions of classical metrics for performance evaluation. Metrics were established considering the genotype mismatch approach recently reported by GA4GH [14]. Metrics were calculated for the three pipelines by using these mathematical definitions, and then the comparison was done. The hap.py pipeline (BaseSpace platform) was implemented for the results of the Isaac variant calling analysis, in a similar way to Krusche et al. [14]. Then we compared the performance metrics for both the hap.py pipeline and our approach (Isaac). Moreover, the visual representation of sets was done using Venn diagram plots, using the Venn-tool, available at http://bioinformatics.psb.ugent.be/webtools/Venn/.

To compare the variant callers performance based on a more comprehensive assessment, an analysis by receiver operating characteristic (ROC) curves was done. Area under the ROC curve (AUC) was calculated and used to determine the statistical significance for each approach and to compare the pipelines The analysis was done using the R software-based easyROC Tool [8] with default parameters. The same program was used to plot the curves, and to calculate statistics for the discrimination ability per algorithm and the pipeline comparison, in both cases using AUC values and a significance level of 95%.

### Annotation of clinically relevant variants

As an example of a specific application of targeted sequencing in the clinical context, the comparison of variant callers was done with ten genes related to HCM [12, 20]. ClinVar database was used for variants annotation [15],National Center for Biotechnology Information, 2018), and InterVar tool for clinical interpretation of genetic variants by the ACMG/AMP 2015 guideline [17]. See Additional file 1: Table S2 “Analysis of variants related to ICCs” for details of the SNPs, genes, and SNP classification by ACGM and ClinVar.

### Running platforms

For all analysis, except Isaac pipeline, homemade scripts were written using python/Unix code and run in the Kabré-CeNaT computational cluster (http://cluster.cenat.ac.cr/). In the case of Isaac pipeline, and hap.py pipeline, the BaseSpace platform was used for running algorithms (https://basespace.illumina.com/dashboard).

## Supplementary Information

**Additional file 1:** Analysis of variants related to ICCs

## Data Availability

The datasets are available at https://basespace.illumina.com/sample/35737707/NA12878. Access by the web-tool or command-line requires an account (free) in the BaseSpace platform. To access by command-line, see instructions here: https://bioinformatics.uconn.edu/data-download-from-basespace-illumina/.

## Abbreviations

ACGM: American College of Medical Genetics and Genomics
ARVC: Arrhythmogenic right ventricular cardiomyopathy
DCM: Dilated cardiomyopathy
FH: Familial hypercholesterolemia
FN: False negative
FP: False positive
GA4GH: Global alliance for genomics and health
GCAT: Genome comparison and analytic testing
GeT-RM: Genetic testing reference materials
GIAB: Genome in a bottle
HCM: Hypertrophic cardiomyopathy
I: Incongruences or discordances
ICCs: Inherited cardiac conditions
NCBI: National Center for Biotechnology Information
NGS: Next generation sequencing
NIST: National Institute of Standards and Technology
O: Out of region of interest
SNP: Single-nucleotide polymorphism
TP: True positive
NAC: Non assessed calls
Ts/Tv ratio: Ratio of transitions/transversions
