# Supplementary material for: Set-theory based benchmarking of three different variant callers for targeted sequencing
Source: BMC Bioinformatics. 2021 Jan 7;22:20. doi: 10.1186/s12859-020-03926-3 (PMC7791862; doi:10.1186/s12859-020-03926-3)
Supplement: Supplementary file 1 — Additional file 1: Analysis of variants related to ICCs [file 12859_2020_3926_MOESM1_ESM.docx]

**Set-theory based benchmarking of three different variant callers for target sequencing**

**Supplementary Material: Analysis of variants related to ICCs**

***Inherited Cardiac Conditions and the TruSight Cardio sequencing kit***

Inherited Cardiac Conditions (ICCs) are a group of cardiovascular diseases with genetic basis. With advances in imaging, clinical procedures, genetics and genomic we now have a major understanding of these diseases (Girolami et al., 2018) and currently, the study of mutations is highly recommended by clinical guidelines.

**Table S1.** Description of some Inherited Cardiac Conditions including in TruSight Cardio Sequencing Panel.

| **Inherited Cardiac Condition** | **Description** | **Related genes** (Celestino-Soper et al., 2017; Pua et al., 2016) |
| --- | --- | --- |
| Dilated Cardiomyopathy (DCM) | DCM is the most common cardiomyopathy, accounting 55% of all cardiomyopathy cases. It is the most heterogeneous disease, with 50% related to a genetic cause with familiar history.  This abnormality causes a diastolic dysfunction with means less myocardial force. Genes related are encoding cytoskeletal, sarcomeric, or Z-disk proteins (Sisakian, 2014; Zorio-grima et al., 2016). | ABCC9, ACTC1, ACTN2, ANKRD1,BAG3,CRYAB,CSRP3,DES, DMD, DTNA, EMD, EYA4, GATAD1, LAMP2, LDB3, LMNA, MYBPC3, MYH7, NEXN, PLN, RAF1, RBM20, SCN5A, SGCD, TAZ, TCAP,TNNC1, TNNI3, TNNT2, TPM1, TTN, TTR, VCL |
| Hypertrophic Cardiomyopathy (HCM) | HCM is characterized by asymmetrical or symmetrical hypertrophy of the left ventricle. HCM clinical presentation results of abnormal diastolic function, with preserved or enhanced global systolic function (Marian, 2010).  It is related with different genes, which encode for sarcomeric proteins: Mutations in the β-myosin heavy chain gene, myosin binding protein C and troponin T are present in a 70-80% of all HCM cases(Sisakian, 2014). | ACTC1, ACTN2,CSRP3, GLA, LAMP2, MYBPC3,MYH7, MYL2, MYL3, MYOZ2, NEXN, PLN,PRKAG2,TNNC1,TNNI3  TNNT2, TPM1, TTR |
| Long QT syndrome (LQTS) | LQTS is characterized by a delayed repolarization of the ventricular myocardium (Tester & Ackerman, 2014).  Approximately 75% of patients with a clinically certain LQTS diagnosis have mutations in genes that encode for ion channel responsible of the cardiac action potential: potassium channel KCNQ1, KCNH2 and sodium channel SCN5A (Channelopathies, Clinical, & Advances, 2017). | KCNE1, KCNE2, KCNH2, KCNJ2, KCNQ1, SCN5A |
| Arrhythmogenic right ventricular cardiomyopathy (ARVC) | In ARVC is characteristic an abnormal desmosome function. ARVC is characterized by cardiomyocytes lost and develop of fibrosis (Quarta, 2016) | DSC2, DSG2, DSP, JUP, LMNA |
| Familial hypercholesterolemia (FH) | FH results in high levels of low-density lipoprotein (LDL) cholesterol. Mutations of the genes encoding for the LDL receptor, apolipoprotein B and proprotein convertase subtilisin/kexin type 9 (PCSK9), are causes for this autosomal dominant inherited condition. It is common that people with this disease develop heart issues before 20s (Vogt, 2015). | APOB, LDLR, PCSK9 |

In this context, TruSight Cardio sequencing kit (Illumina), a panel of 174 critical genes related to ICCs, can be used to evaluates genetic predisposition for cardiomyopathies, arrhythmias, aortopathies and others (Illumina, 2016). Table S1 summarizes description and genes associated to some of these cardiopathies.

After the benchmarking of variant callers was done in our approach, we selected those variants belonging to Hypertrophic Cardiomyopathy (HCM) genes, as detailed below.

***Annotation of identified variants in Hypertrophic Cardiomyopathy (HCM) genes***

For variant annotation of true variants (TP), we selected ten genes related with HCM as example. HCM this is the most common inherited cardiovascular disease, and it has clinical heterogeneity presentation, most of them related to sudden cardiac death (Puckelwartz & McNally, 2017). After analysis and annotation, the three pipelines achieved identification of variants in three genes related to HCM, as shown in Table S2. All variants were classified as benign or likely benign variants, as expected due we are using the NA12878 genome and not a genome from a case of cardiomyopathy.

**Table S2.** Description of true variants in genes related to Hypertrophic Cardiomyopathy, genome NA12878.

| **Gene** | **SNP** | **Clinical significance** | | **Description** |
| --- | --- | --- | --- | --- |
|  |  | **ACGM** | **Clin Var** |  |
| TPM1 | rs1071646 | Benign | Benign/Likely benign | This mutation in c.688G>A produce a protein amino acid change p.Asp230Asn. In a Chinese study, the detection of SNP loci in the TPM1 suggest that this variation could involve in the pathogenesis of DCM and HCM although the mechanisms remain unclear(Duzkale, Shen, & McLaughlin, 2013; Y. D. Li et al., 2015) |
| TNTT2 | rs3729547 | Benign/Likely benign | Likely benign, Uncertain significance | Missense mutation c.318C>G. it produce an amino acid change p.Ile106Met. No more clinical information provided (Landrum et al., 2018; Rani et al., 2012) |
|  | rs3729845 | Benign/Likely benign | Benign | Mutation at the nucleotide position g.13011. Transition nucleotide A>G. In a DCM study with Indian population, about 4% of heterozygous genotype was observed in DCM, but it was completely absent in the controls of two different population (Y. D. Li et al., 2015). |
|  | rs45520032 | Likely benign | Likely benign, Uncertain significance, conflictive | Missense variant, c.662T>C. No more clinical information provided (Landrum et al., 2018)(Landrum et al., 2018). |
| MYH7 | rs2069540 | Benign | Benign. Gene encodes for β-myosin heavy chain (MYH7), gene that carries most of HCM-related mutations (Vandenberg, Perry, & Hill, 2017). | Synonymous mutation in position g.4902592G >A (Dames, Durtschi, Geiersbach, Stephens, & Voelkerding, 2010). |
|  | rs7157716 | Benign |  | Synonymous mutation in position g.4892727A>G (Dames et al., 2010). |
|  | rs735711 | Benign |  | Synonymous mutation in position g.4898866C>T (Dames et al., 2010). |

The variant in *TPM1,* coding for tropomyosin 1, was classified as benign or likely benign; some studies suggest that this gene could be involved in pathogenesis of DCM and HCM (Sisakian, 2014). *TNNT2* gene codes for the cardiac troponin T and it is found solely in the cardiac muscle. No pathogenic variants were identified. Same significance was found for variants in *MYH7* gene, coding for the heavy chain of slow/beta‐cardiac myosin (Q. Li & Wang, 2017; Sisakian, 2014).

**Table S3.** Description of False positives or Incongruent variants identified by pipelines

| **Type** | **Variant caller** | **SNP** | **Chr** | **gene** | **Clinical Significance** | **Phenotypes** |
| --- | --- | --- | --- | --- | --- | --- |
| False positives | VarScan | rs187423999 | 10 | RBM20 | Conflicting interpretations | Dilated cardiomyopathy 1DD,Not specified, dilated Cardiomyopathy, Dominant |
|  |  | rs45579241 | 10 | MAP2K2 | Benign | Myofibrillar myopathy, ZASP-related |
|  |  | rs121918470 | 12 |  | Pathogenic | LEOPARD syndrome 1, Rasopathy |
|  |  |  | 12 |  | Pathogenic | Noonan syndrome 1 |
|  |  | rs144383241 | 19 | MAP2K2 | Benign/Likely benign | Rasopathy |
|  |  | rs150486780 | 1 | CASQ2 | Likely benign | Not specified |
|  |  | rs148824162 | 1 |  | Conflicting interpretations | Not specified |
|  |  | rs61233923 | 2 | TTN | Benign | Not specified |
|  |  | rs143154982 | 2 | DES | Likely benign | Not specified |
|  |  | rs116840786 | 3 | ACTN2 | Pathogenic | Creatine phosphokinase, elevated serum |
|  |  | rs147078770 | 6 | LAMA4 | Likely benign | Dilated cardiomyopathy 1JJ,Not specified |
|  |  | rs201046790 | X | TAZ | Likely benign | Left ventricular non compaction cardiomyopathy, endocardial fibroelastosis, 3-Methylglutaconic aciduria type 2, dilated cardiomyopathy 3B |
|  |  | rs201808867* | 10 | VCL | Unknown | Dilated cardiomyopathy |
|  |  | rs76982592* | 12 | PTPN12 |  | Leukemia |
|  |  | rs142271248* | 19 | MAP2K2 | Unknown | CFC Syndrome |
|  |  | rs12913013* | Not available information | | | |
|  | VarScan and Freebayes | rs73219144 | X | LAMP2 | Benign/Likely benign | Hypertrophic cardiomyopathy,Danon disease,Not specified,Cardiovascular phenotype |
|  |  | rs12097 | X |  | Benign/Likely benign | Hypertrophic cardiomyopathy,Danon disease,Not specified,Cardiovascular phenotype |
|  |  | rs3729711 | 19 | TNNI3 | Conflicting interpretations | Cardiomyopathy,Not specified,not provided |
|  |  |  |  |  | Benign/Likely benign | Hypertrophic cardiomyopathy,Primary ciliary dyskinesia,Not specified,Cardiovascular phenotype |
| Incongruences | Freebayes | rs9387061 | 6 | LAMA4 | Benign | Not specified |
|  |  | rs9400522 | 6 | LAMA4 | Benign | Not specified |
|  | VarScan | rs1341864 | 1 | ACTN2 | Benign | Primary familial hypertrophic cardiomyopathy, dilated cardiomyopathy 1AA |
|  |  |  | 1 | ACTN2 | Benign | Not specified |
|  |  | [rs1051375](http://genome.ucsc.edu/cgi-bin/hgTracks?db=hg19&position=chr12%3A2788878-2788879) | 12 | [CACNA1C](https://www.ncbi.nlm.nih.gov/entrez/query.fcgi?db=gene&cmd=Retrieve&dopt=Graphics&list_uids=775) | Benign | Brugada syndrome, Timothy syndrome, Cardiovascular phenotype |

***Annotation of False positives or Incongruent variants identified by pipelines***

We analyzed variants which were classified as FP or Incongruences, as shown in Table S3. In the case of FP, 18 variants were identified by VarScan, some of them related to cardiovascular syndromes. Three of these variants are known to be pathogenic for cardiovascular diseases, and the rest of them were classified as benign or with conflictive interpretation. Three of the FP by VarScan were also identified as FP using Freebayes in *LAMP2* and *TNNI3* genes, both related with HCM or DCM phenotypes.

On the other hand, Freebayes had two I variants in *LAMA4* gene, which has not a specific phenotype association. VarScan identified three inconsistent variants in *ACTN2* gene, both related with cardiomyopathies. A third I element was a variant in *CACNA1C* gene, encoding for a voltage dependent calcium channel and it would be related to cardiovascular phenotype. All these variants identified as incongruences resulted as benign according to ClinVar classification.

**References**

Celestino-Soper, P. B. S., Gao, H., Lynnes, T. C., Lin, H., Liu, Y., Spoonamore, K. G., … Vatta, M. (2017). Validation and Utilization of a Clinical Next-Generation Sequencing Panel for Selected Cardiovascular Disorders. *Frontiers in Cardiovascular Medicine*, *4*(March). https://doi.org/10.3389/fcvm.2017.00011

Channelopathies, C., Clinical, R., & Advances, G. (2017). Cardiac Channelopathies and Sudden Death : Recent Clinical and Genetic Advances, (Dcm), 1–21. https://doi.org/10.3390/biology6010007

Dames, S., Durtschi, J., Geiersbach, K., Stephens, J., & Voelkerding, K. V. (2010). Comparison of the illumina genome analyzer and roche 454 GS FLX for resequencing of hypertrophic cardiomyopathy-associated genes. *Journal of Biomolecular Techniques*, *21*(2), 73–80.

Duzkale, H., Shen, J., & McLaughlin, H. (2013). A systematic approach to assessing the clinical significance of genetic variants. *… Genetics*. https://doi.org/10.1002/cge.12257

Girolami, F., Frisso, G., Benelli, M., Crotti, L., Iascone, M., Mango, R., … Basso, C. (2018). Contemporary genetic testing in inherited cardiac disease : tools , ethical issues , and clinical applications, *0*. https://doi.org/10.2459/JCM.0000000000000589

Illumina. TruSight ® Cardio Sequencing Kit (2016). Retrieved from https://basespace.illumina.com/dashboard

Landrum, M. J., Lee, J. M., Benson, M., Brown, G. R., Chao, C., Chitipiralla, S., … Maglott, D. R. (2018). ClinVar: improving access to variant interpretations and supporting evidence. *Nucleic Acids Research*, *46*(D1), D1062–D1067. https://doi.org/10.1093/nar/gkx1153

Li, Q., & Wang, K. (2017). InterVar : Clinical Interpretation of Genetic Variants by the 2015 ACMG-AMP Guidelines, 267–280. https://doi.org/10.1016/j.ajhg.2017.01.004

Li, Y. D., Ji, Y. T., Zhou, X. H., Li, H. L., Zhang, H. T., & Zhang, Y. (2015). Significance of sarcomere gene mutation in patients with dilated cardiomyopathy, *2*(3), 11200–11210.

Marian, A. J. (2010). Hypertrophic cardiomyopathy: From genetics to treatment. *European Journal of Clinical Investigation*, *40*(4), 360–369. https://doi.org/10.1111/j.1365-2362.2010.02268.x

Pua, C. J., Bhalshankar, J., Miao, K., Walsh, R., John, S., Lim, S. Q., … Cook, S. A. (2016). Development of a Comprehensive Sequencing Assay for Inherited Cardiac Condition Genes, 3–11. https://doi.org/10.1007/s12265-016-9673-5

Puckelwartz, M. J., & McNally, E. M. (2017). Hypertrophic Cardiomyopathy Gene Testing. *Circulation: Cardiovascular Genetics*, *10*(5), 2–4. https://doi.org/10.1161/CIRCGENETICS.117.001951

Quarta, G. (2016). Criteria for Arrhythmogenic Right Ventricular Cardiomyopathy. *Revista Española de Cardiología*, *65*(7), 599–605. https://doi.org/10.1016/j.recesp.2012.02.016

Rani, D. S., Nallari, P., Priyamvada, S., Narasimhan, C., Singh, L., & Thangaraj, K. (2012). High prevalence of Arginine to Glutamine Substitution at 98, 141 and 162 positions in Troponin I (TNNI3) associated with hypertrophic cardiomyopathy among Indians. *BMC Medical Genetics*, *13*, 1–8. https://doi.org/10.1186/1471-2350-13-69

Sisakian, H. (2014). Cardiomyopathies: Evolution of pathogenesis concepts and potential for new therapies. *World Journal of Cardiology*, *6*(6), 478. https://doi.org/10.4330/wjc.v6.i6.478

Tester, D. J., & Ackerman, M. J. (2014). Genetics of Long Qt Syndrome. *Methodist DeBakey Cardiovascular Journal*, *10*(1), 29–33.

Vandenberg, J. I., Perry, M. D., & Hill, A. P. (2017). Recent advances in understanding and prevention of sudden cardiac death [ version 1 ; referees : 2 approved ] Referee Status :, *6*(0), 1–7. https://doi.org/10.12688/f1000research.11855.1

Vogt, A. (2015). The genetics of familial hypercholesterolemia and emerging therapies. *Application of Clinical Genetics*, *8*, 27–36. https://doi.org/10.2147/TACG.S44315

Zorio-grima, E., Barriales-villa, R., Ramo, J., Evangelista-masip, A., Moya-mitjans, A., Serratosa-ferna, L., … Albert-brotons, D. C. (2016). Cardiopatías familiares: Protocolo de actuación. *Revista Espanola de Cardiologia*, *69*(x), 300–309.
